# Supplementary material for: Structure of human CALHM1 reveals key locations for channel regulation and blockade by ruthenium red
Source: Nat Commun. 2023 Jun 28;14:3821. doi: 10.1038/s41467-023-39388-3 (PMC10307800; doi:10.1038/s41467-023-39388-3)
Supplement: Supplementary file 1 — Supplementary Information [file 41467_2023_39388_MOESM1_ESM.pdf]

Supplementary Information for

Structure of human CALHM1 reveals key locations for channel regulation and blockade by ruthenium red

Johanna L Syrjänen[1], Max Epstein[1], Ricardo Gómez[1], Hiro Furukawa[1, *]

[1] W.M. Keck Structural Biology Laboratory, Cold Spring Harbor Laboratory, Cold Spring Harbor, New York 11724, USA.

Supplementary Figures

Supplementary Figure 1. Single-particle analysis of chCALHM1Δct proteins expressed in HEK293 cells
Supplementary Figure 2. Single-particle analysis of hCALHM1Δct proteins expressed in HEK293 cells
Supplementary Figure 3. ChCALHM1Δct cryo-EM structure determined from proteins expressed in HEK293 cells and highlights of key regions in hCALHM1Δct
Supplementary Figure 4. Location of lipid density and assessment of CG-MD convergence
Supplementary Figure 5. Representative traces for the whole-cell patch-clamp recordings of human CALHM1Δct and point mutants
Supplementary Figure 6. Single-particle analysis of hCALHM1$_{I109W}$Δct in the presence of RuR
Supplementary Figure 7. Local resolution calculations and representative densities of hCALHM1$_{I109W}$Δct in the presence of RuR
Supplementary Figure 8. The cryo-EM structure of hCALHM1$_{I109W}$Δct with RuR (c1 reconstruction)
Supplementary Figure 9. Single-particle analysis of hCALHM1I109WΔct in the absence of RuR
Supplementary Figure 10. The cryo-EM structure of hCALHM1$_{I109W}$Δct in the absence of RuR
Supplementary Figure 11. Structure comparisons of hCALHM1Δct, hCALHM1$_{I109W}$Δct and RuRhCALHM1$_{I109W}$Δct structures
Supplementary Figure 12. Assessment of CG-MD PMF calculation convergence in NTH containing hCALHM1 $_{I109W}$Δct and hCALHM1Δct and unbiased CG-MD POPC binding mode.

Supplementary Tables

Supplementary Table 1. Cryo-EM data collection, refinement and validation statistics
Supplementary Table 2. Summary of number of experiments, current density, statistical tests and p values for experiments shown in Figure 3b.
Supplementary Table 3. Summary of number of experiments, normalized current amplitude, statistical tests and p values for experiments shown in Figure 6b.
Supplementary Table 4. Summary of number of experiments, normalized current amplitude, statistical tests and p values for experiments shown in Figure 6d.

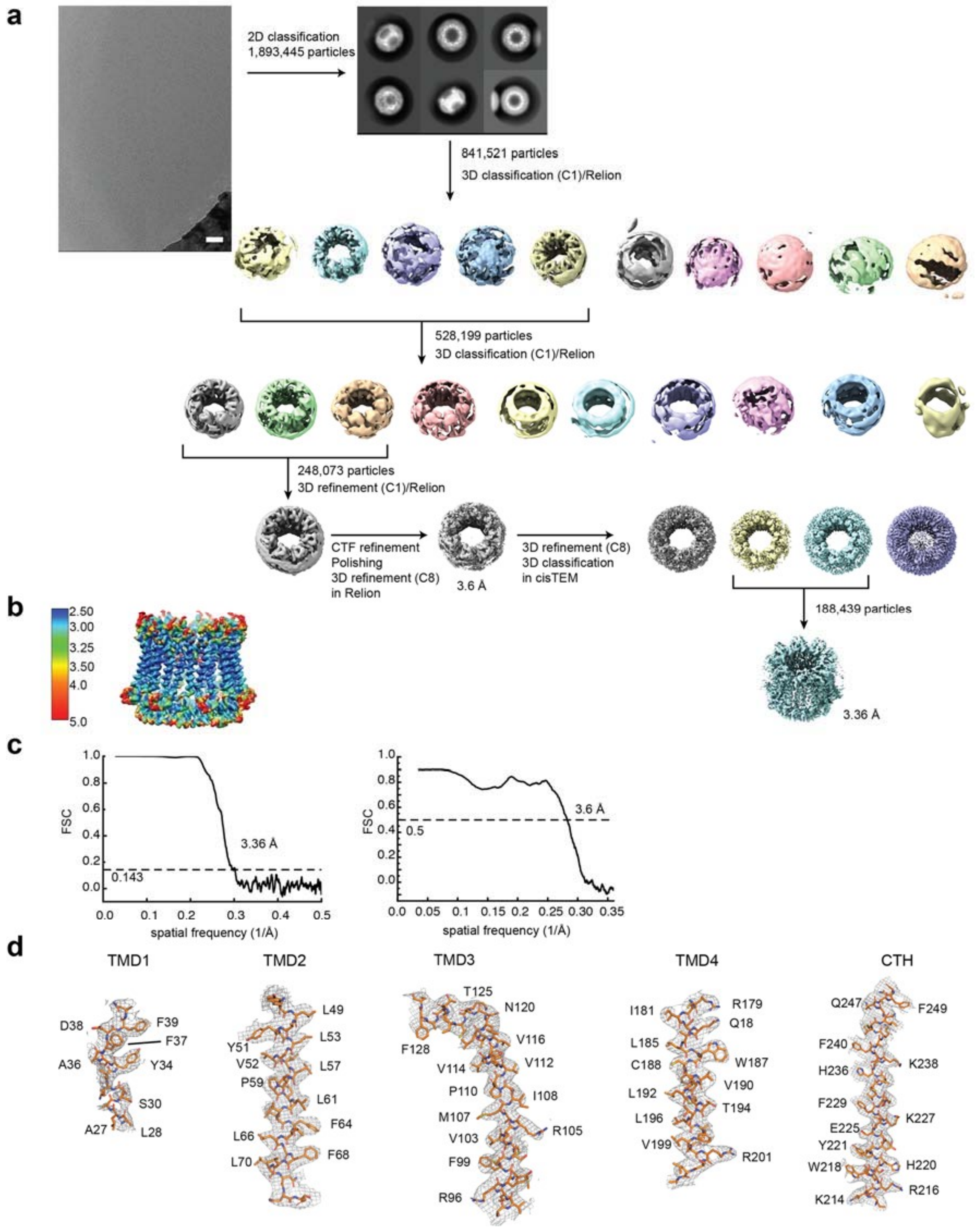

Supplementary Figure 1. Single-particle analysis of chCALHM1Δct proteins expressed in HEK293 cells. a A representative micrograph, representative 2D classes and the 3D classification workflow are shown. The scale bar corresponds to 35 nm. **b** Local resolutions were calculated using ResMap. The scale is in Å. **c** The FSC plots of the two half maps (left) and the map vs. model (right). **d** Cryo-EM densities of TMD1-4 and the CTH are shown.

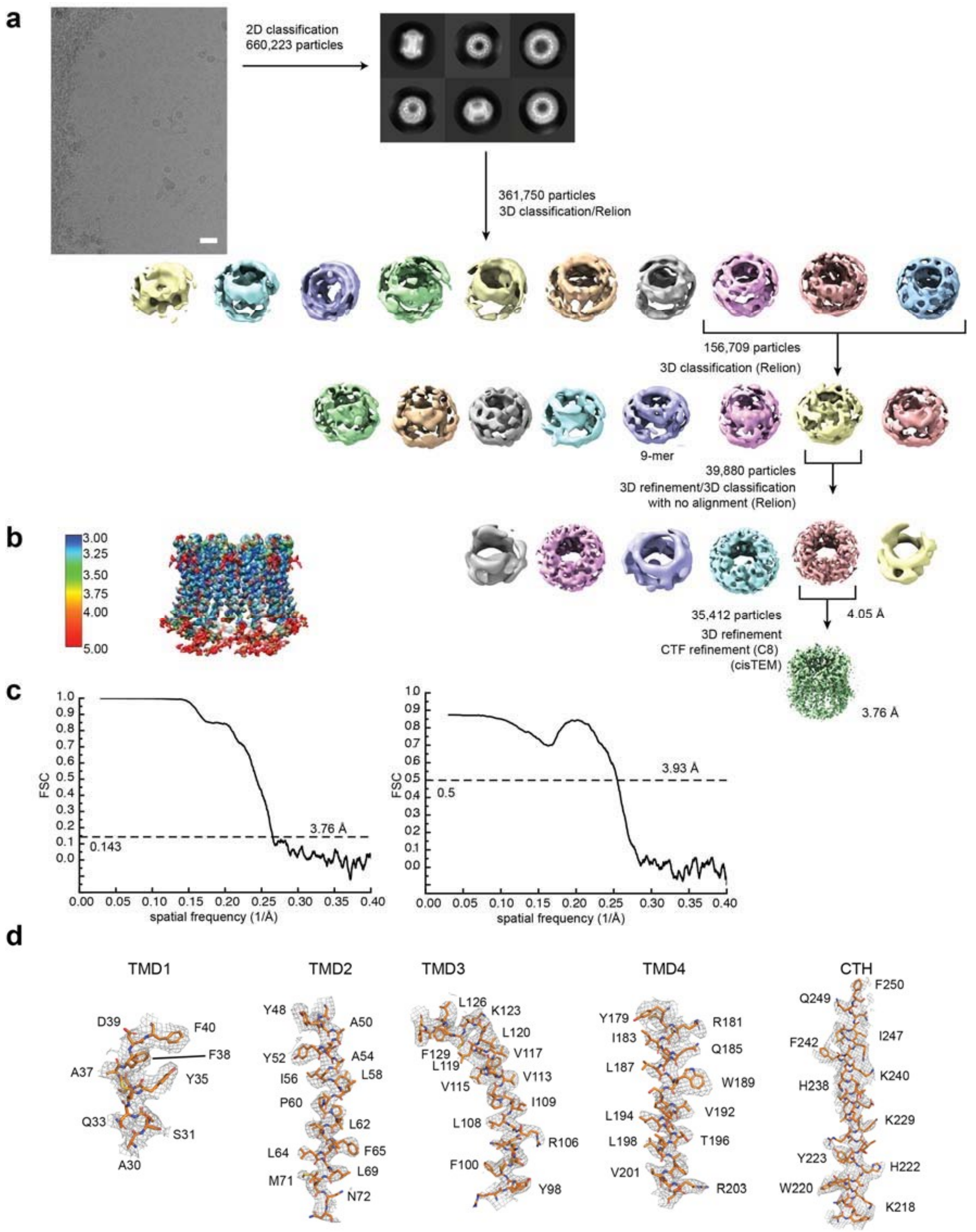

Supplementary Figure 2. Single-particle analysis of hCALHM1∆ct proteins expressed in HEK293 cells. a A representative micrograph, representative 2D classes and the 3D classification workflow are shown. The scale bar corresponds to 35 nm. **b** Local resolutions were calculated using ResMap**.** The scale is in Å**. c** The FSC plots of the two half maps (left) and the map vs. model (right). **d** Cryo-EM densities of TMD1-4 and the CTH are shown.

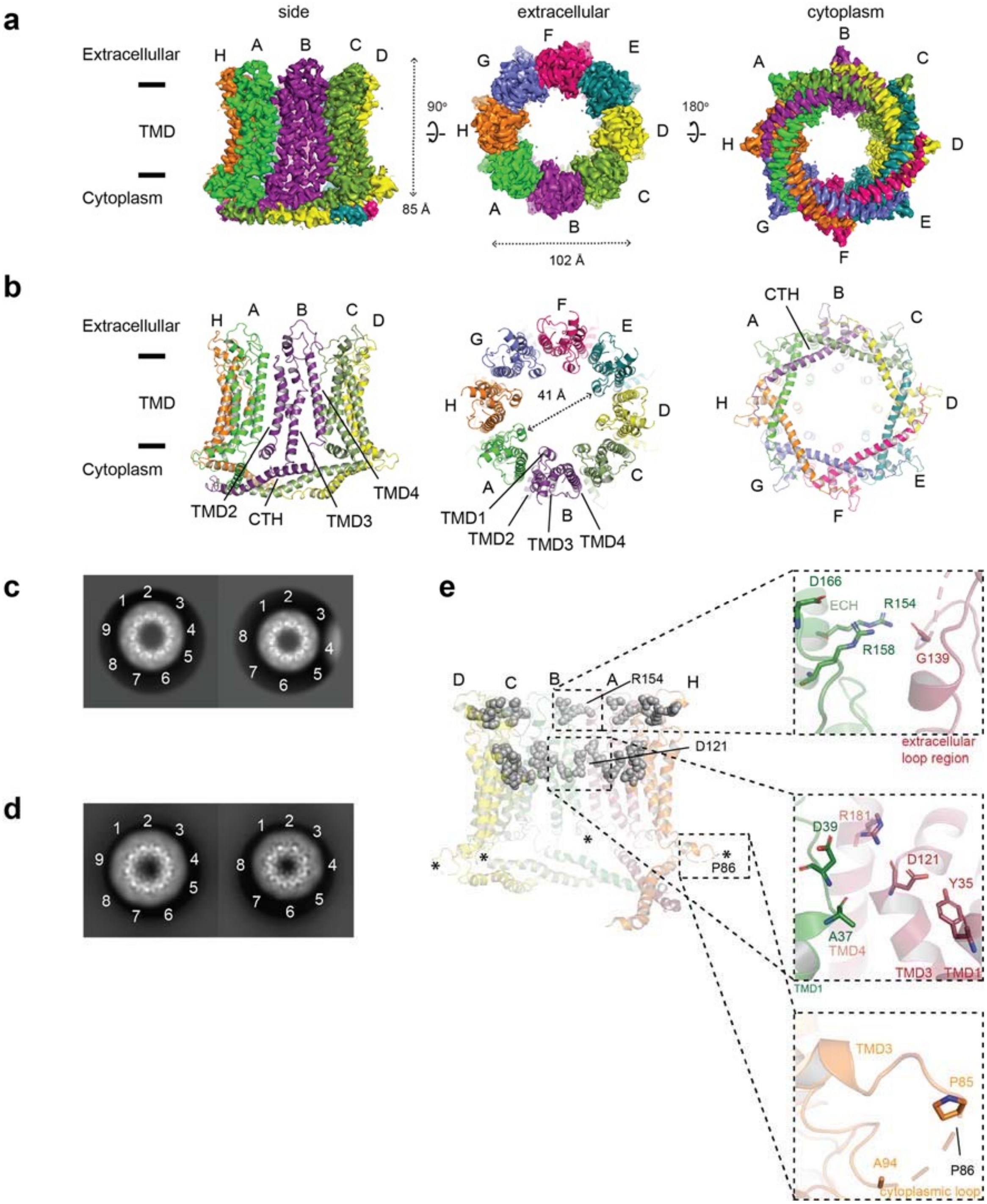

Supplementary Figure 3. chCALHM1∆ct cryo-EM structure determined from proteins expressed in HEK293 cells and highlights of key regions in hCALHM1∆ct. a Cryo-EM density and **b** an atomic model of chCALHM1∆ct viewed from the side of the membrane, the extracellular region and the cytoplasm (c8 symmetry). The transmembrane helices TMD1-4 are labelled, as well the C-terminal helix CTH. Modeled regions include the loop between TMD2 and TMD3 (residues 85-90), part of the extracellular domain (residues 139-145) and an extended C-terminus (residues 249-260), which were not resolved in PDB-6VAM/EMD-21143. The pore

distance indicated by the double ended arrow is measured between the Gln33 Cα positions of chains A and E. **c** 2D classification of chCALHM1∆ct reveals octameric and nonameric species. **d** Analysis of EMPIAR #10444 (killifish CALHM1) also reveals octameric and nonameric species. **e** Locations of functionally important residues can be mapped in the human and chicken CALHM1Δct structures. A side view of human CALHM1Δct with residues indicated by space filled models or asterisks. Insets depict zoomed in views of Arg154 (top); Asp121 (middle); and the location of Pro86 (bottom).

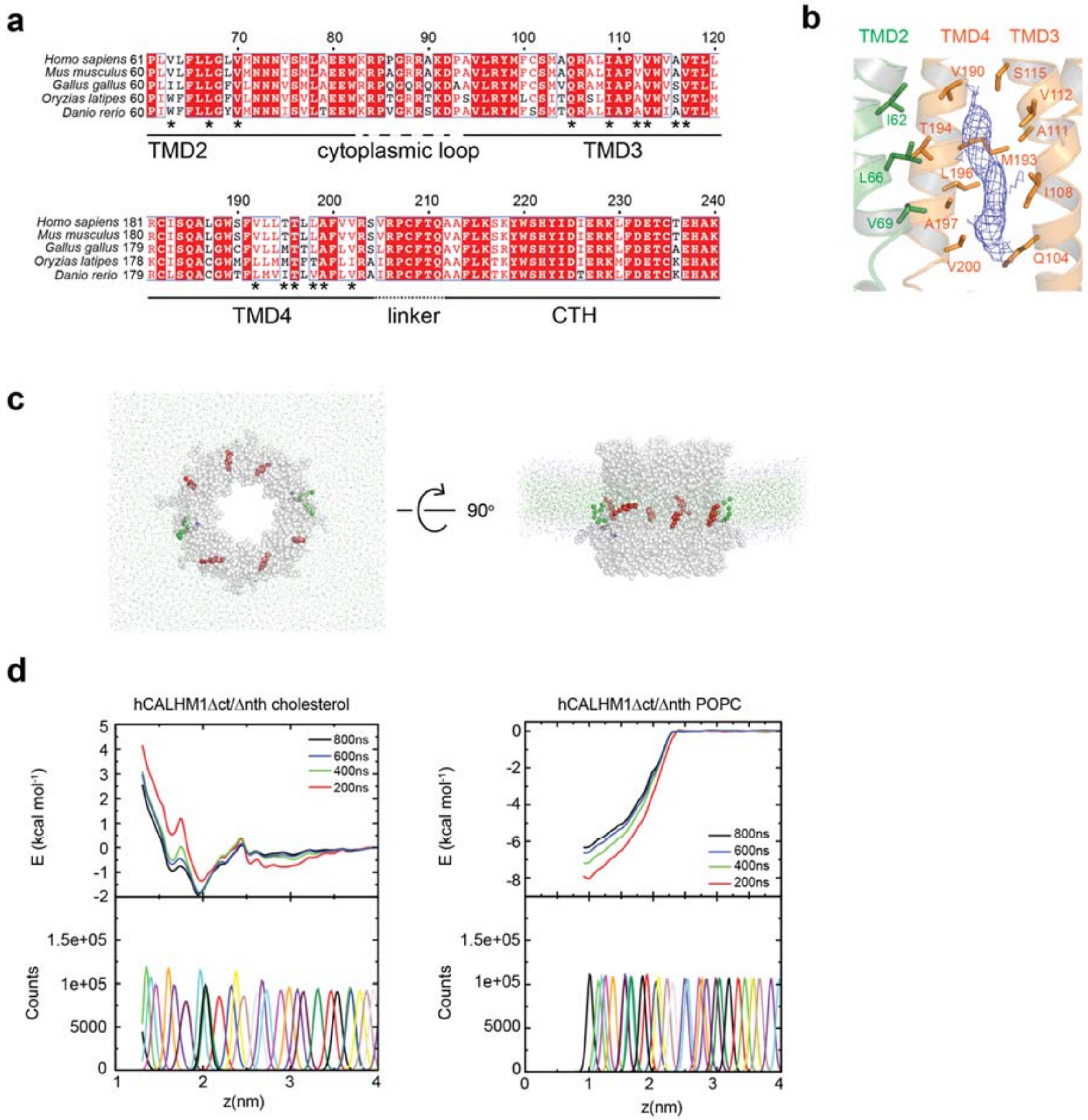

Supplementary Figure 4. Location of lipid density and assessment of CG-MD convergence. a A sequence alignment of human, mouse, chicken, killifish, and zebrafish CALHM1 encompassing the regions of the protein that form the mostly hydrophobic pocket. Residues forming the pocket are indicated by asterisks. **b** Lipid-like density can be observed within the hydrophobic pocket of chicken CALHM1Δct purified from mammalian HEK293 GnTI⁻ cells. **c** Representative snapshot from unbiased CG-MD simulation of hCALHM1 Δct/Δnth (grey transparent beads) with bound POPC (green, grey and blue beads) and cholesterol (red) molecules that freely diffused into the binding site over the course of the simulation. Both a top-down cytoplasmic view and side-on view lateral to the phospholipid bilayer are shown. POPC orientation is consistent for all binding events where the charged PC headgroup points towards the solvated pore and alkyl tails point towards the membrane, residing in the cytoplasmic leaflet.

This formed the basis for selecting the starting conformation of POPC in atomistic simulations. **d** Block analysis of cholesterol (left panel) and POPC (right panel) of 200-400 ns (red), 200-600 ns (green), 200-800 ns (blue), 200-1000 ns (black) demonstrating convergence to within thermal energy for both systems. Associated individual umbrella histograms show good spacing and overlap along both reaction coordinates. The amino-terminal helices (NTHs) were not included in these MD models since they were not resolved in the hCALHM1Δct cryo-EM structure.

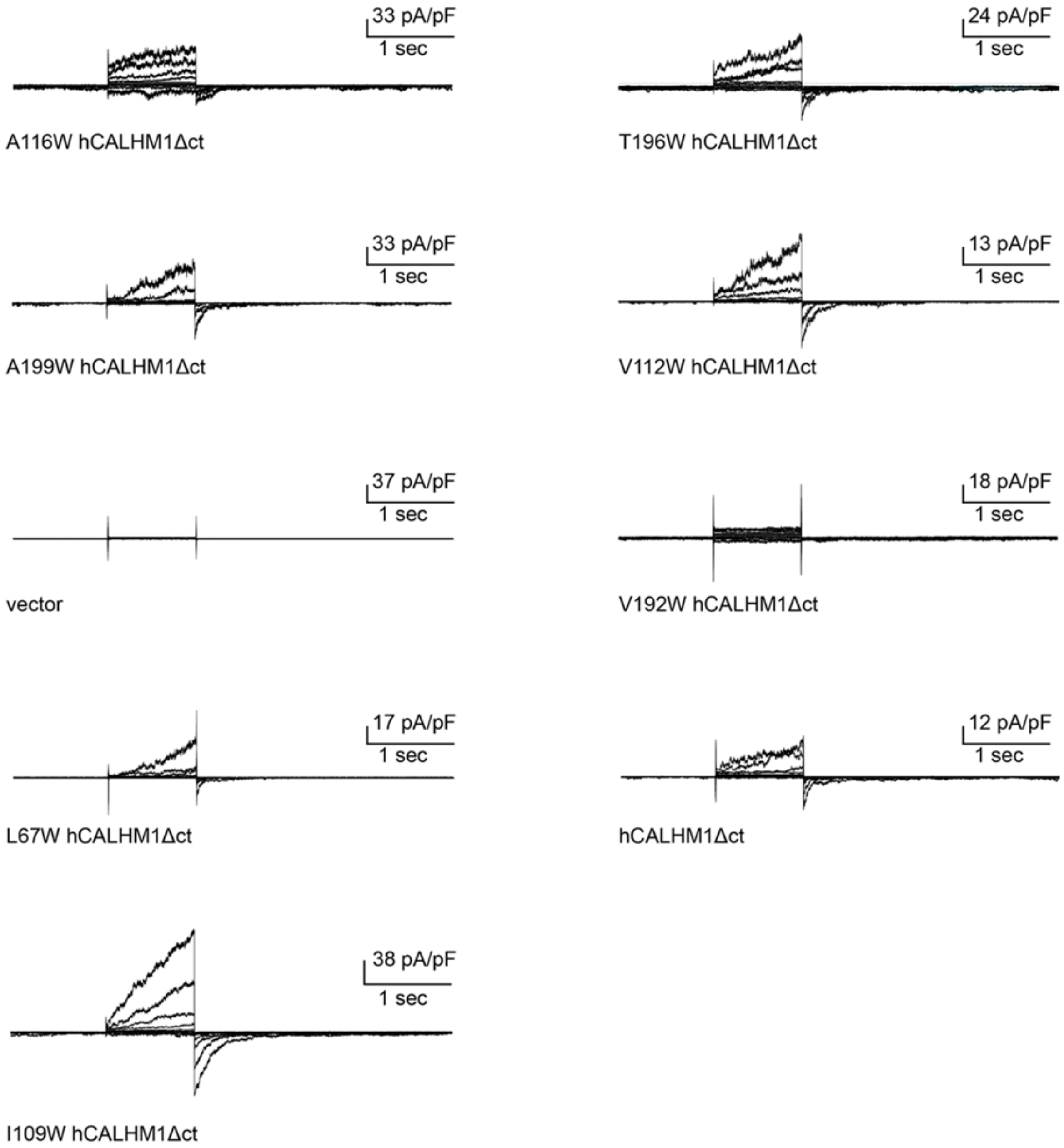

Supplementary Figure 5. Representative traces for the whole-cell patch-clamp recordings of human wild-type CALHM1Δct and point mutants. In each case, the voltage was clamped at -60mV and then stepped to a different voltage per sweep. The voltage steps ranged from 100 to +100 mV and were increased in increments of 20 mV. The cell was held at -60mV for three seconds before the next sweep.

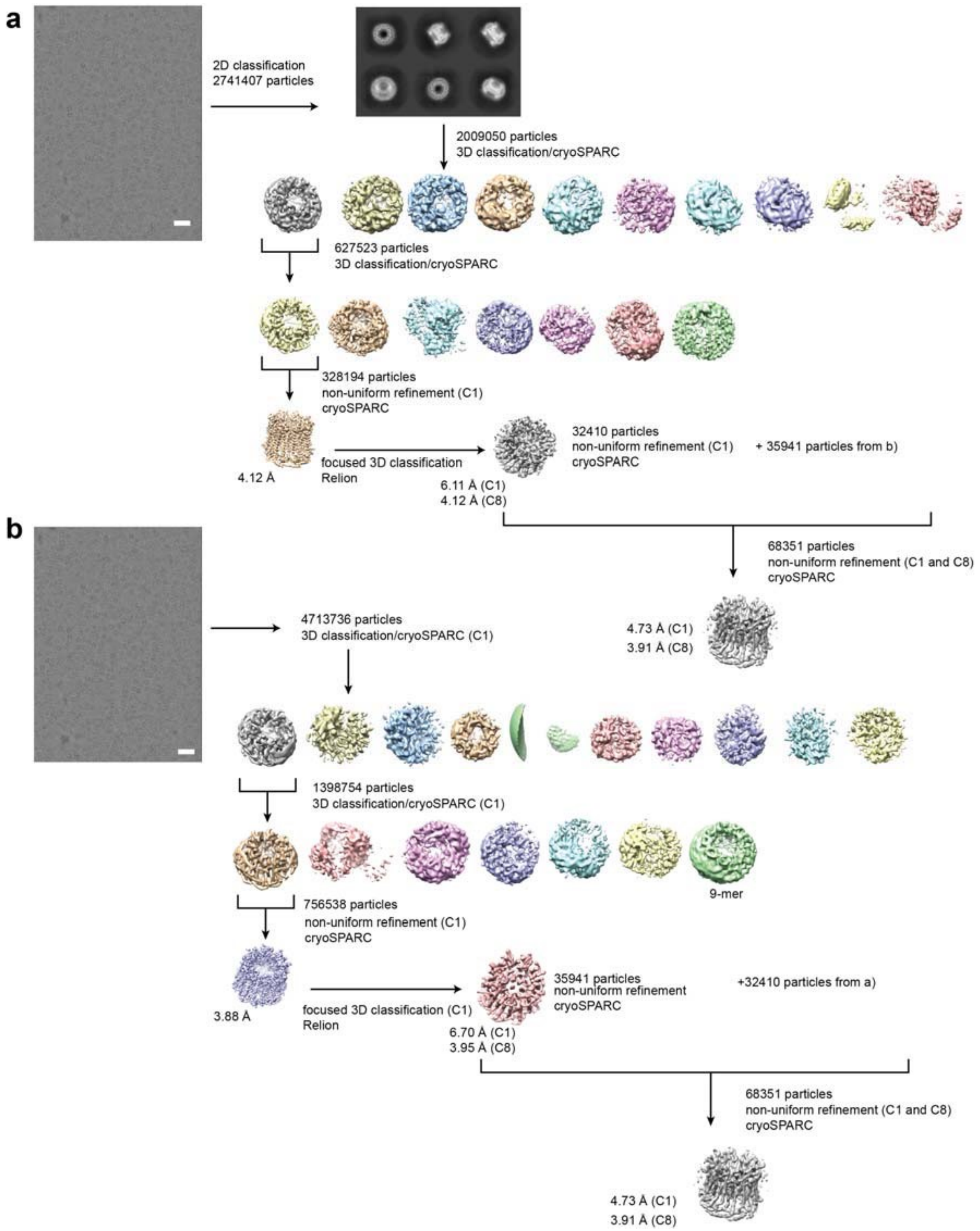

Supplementary Figure 6. Single-particle analysis of hCALHM1$_{I109W}\Delta$ct in the presence of RuR.
a and **b** Representative micrographs, representative 2D classes and the 3D classification workflows are shown. The final maps are merged from two datasets as indicated. The scale bar in the micrograph corresponds to 35 nm.

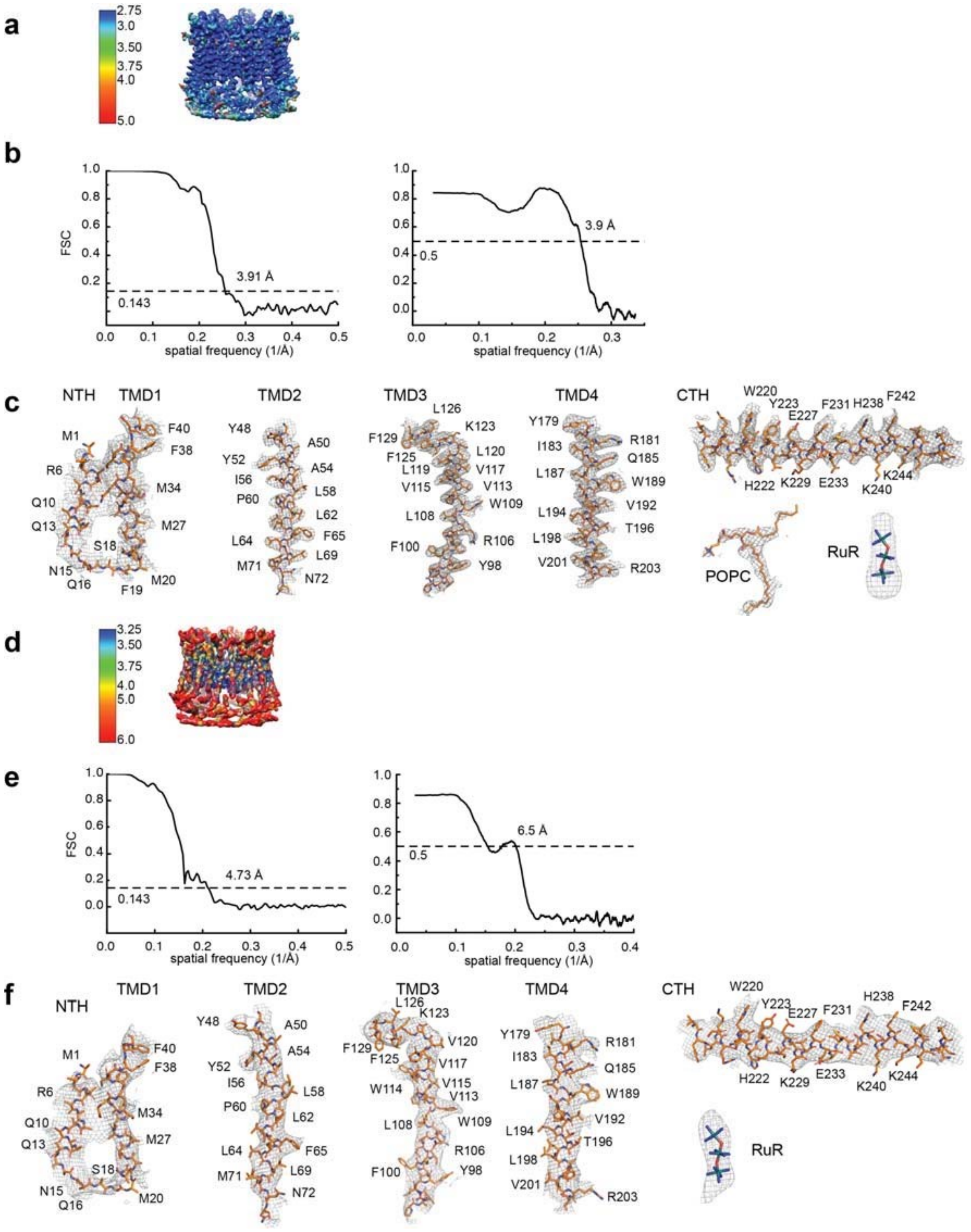

Supplementary Figure 7. Local resolution calculations and representative densities of hCALHM1$_{\mathrm{I109W}}\Delta$ct in the presence of RuR. **a** Local resolutions of the C8 hCALHM1$_{\mathrm{I109W}}\Delta$ct map were calculated using ResMap. **b** The FSC plots of the two half maps (left) and model vs. map (right) for the C8 data. **c** Cryo-EM densities of TMD1-NTH, TMD2-4, the CTH, lipid and RuR are shown, with C8 symmetry. **d** Local resolutions of the C1 hCALHM1$_{\mathrm{I109W}}\Delta$ct map were calculated using ResMap. The scale is in Å. **e** The FSC plots of the two half maps (left) and model vs. map

(right), without imposing symmetry (C1). **f** Cryo-EM densities of TMD1-NTH, TMD2-4, the CTH, lipid and RuR are shown, without imposing symmetry (C1).

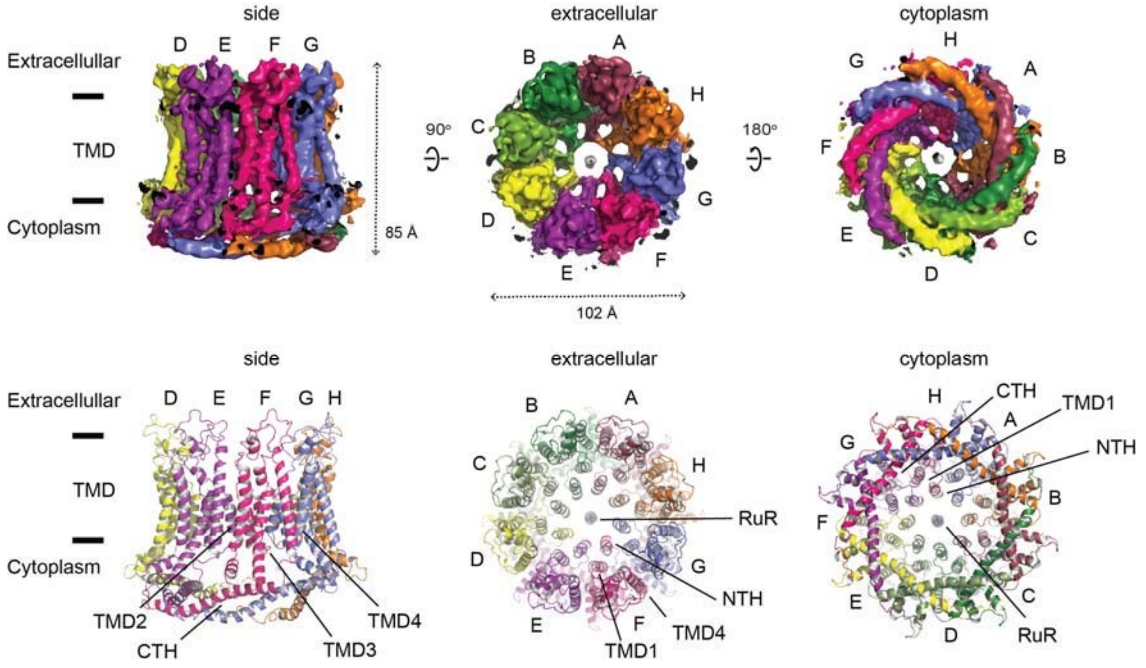

Supplementary Figure 8. The cryo-EM structure of hCALHM1$_{I109W}\Delta$ct with RuR (C1 reconstruction). Cryo-EM density (top) and an atomic model of human hCALHM1$_{I109W}\Delta$ct-RR (bottom) viewed from the side of the membrane, the extracellular region and the cytoplasm (C1 reconstruction).

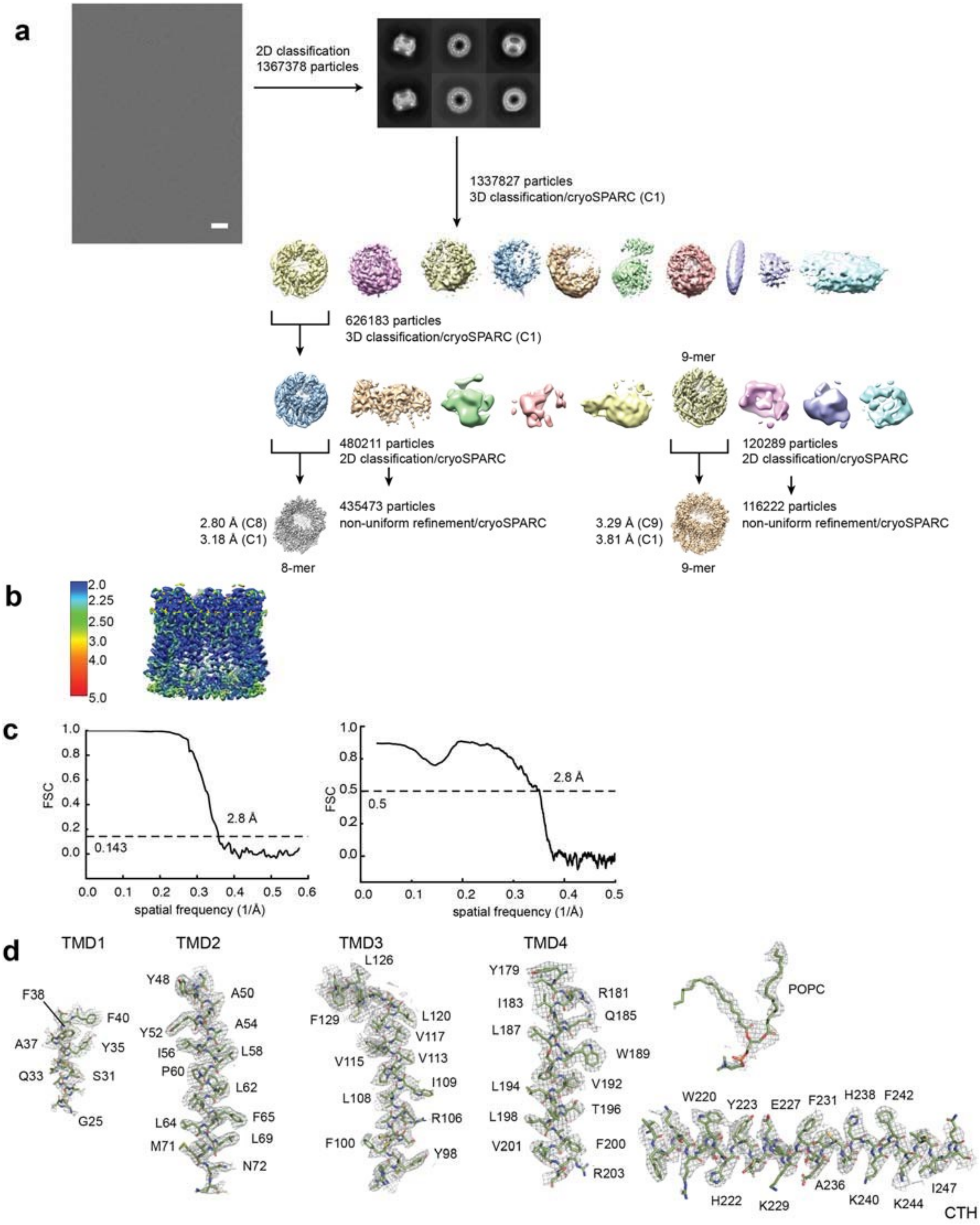

Supplementary Figure 9. Single-particle analysis of hCALHM1I109WΔct in the absence of RuR. **a** Representative micrographs, representative 2D classes and the 3D classification workflows are shown. The scale bar corresponds to 35 nm. **b** Local resolutions of the hCALHM1I109WΔct map were calculated using ResMap. The scale is in Å. **c** The FSC plots of the two half maps (left) and model vs. map (right). **d** Cryo-EM densities of TMD1, TMD2-4, the CTH, and lipid are shown.

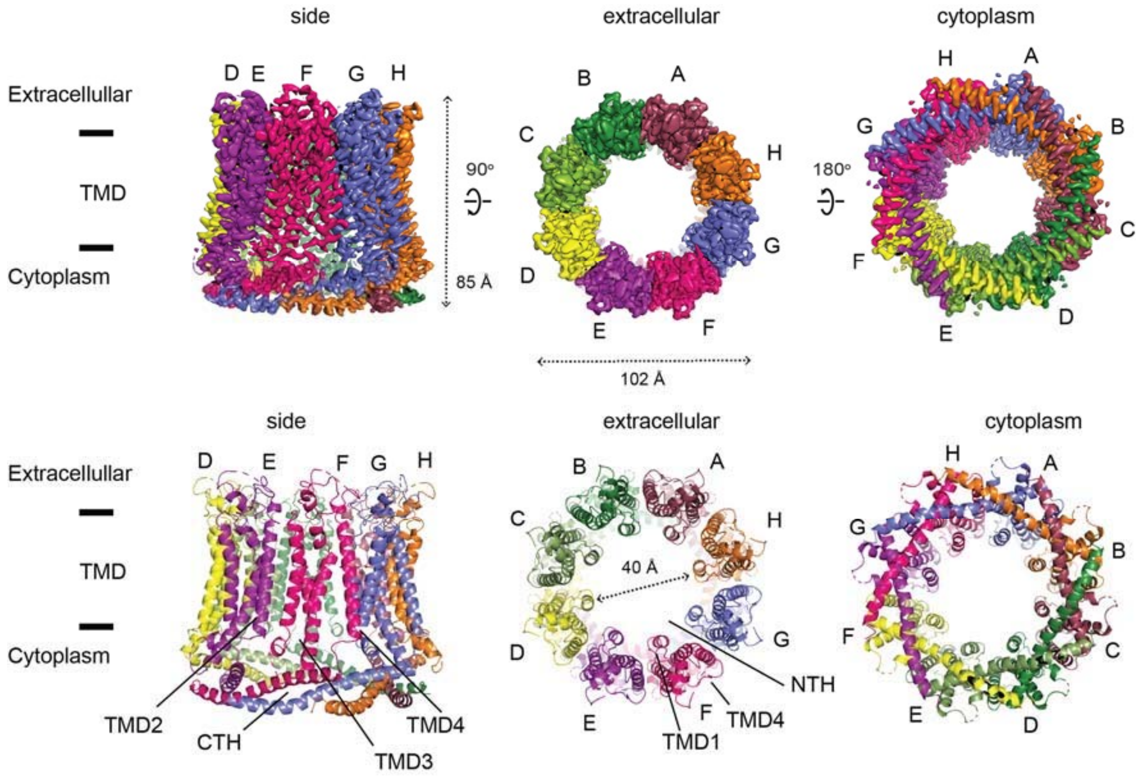

Supplementary Figure 10. The cryo-EM structure of hCALHM1$_{I109W}$Δct in the absence of RuR. Cryo-EM density (top) and an atomic model of human hCALHM1$_{I109W}$Δct (bottom) viewed from the side of the membrane, the extracellular region and the cytoplasm (c8 symmetry). The pore distance indicated by the double-ended arrow is measured between the Gln33 Cα positions of chains D and H.

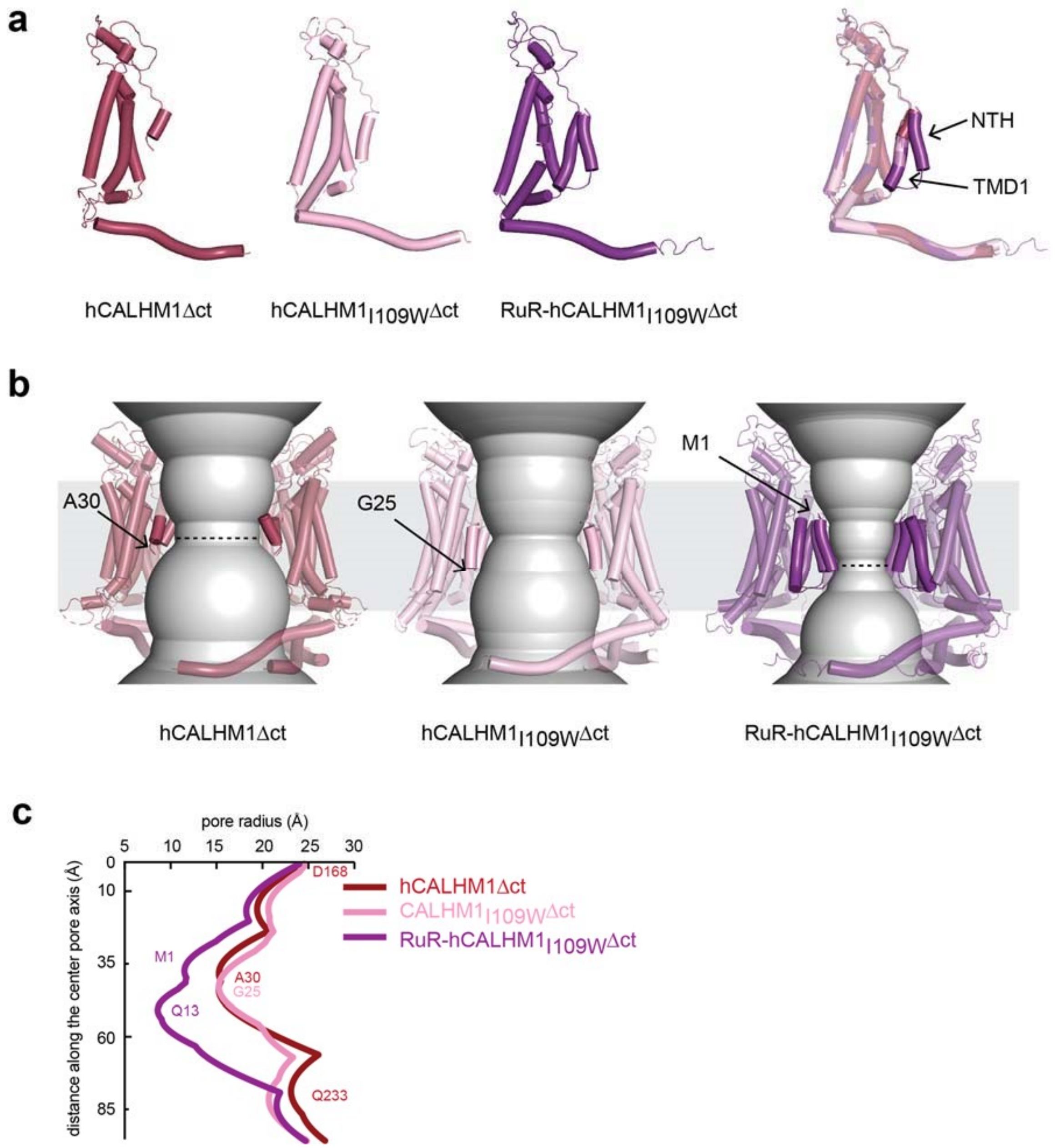

Supplementary Figure 11. Structure comparisons of hCALHM1Δct, hCALHM1$_{I109W}$Δct and RuR-hCALHM1$_{I109W}$Δct. a Protomers of hCALHM1Δct (raspberry), hCALHM1$_{I109W}$Δct (light pink) and RuR-hCALHM1$_{I109W}$Δct (violet purple) are shown. An overlay of the three protomers is shown on the right hand side. Arrows indicate the positions of the N-terminal helix (NTH) and the first transmembrane domain (TMD1). **B** The channel pores according to models of the resolved cryo-EM density of hCALHM1Δct (raspberry), hCALHM1$_{I109W}$Δct (light pink) and RuRhCALHM1$_{I109W}$Δct (violet purple) are shown. The most N-terminal residues that can be modeled in each case are indicated. The dashed line indicates the pore distance measured between the Gln33 Cα positions of apposing chains of hCALHM1Δct (corresponding to ~41 Å) or between the Gln13 Cα positions of apposing chains of RuR-hCALHM1$_{I109W}$Δct (corresponding

to ~23 Å). **c** A comparison of the pore radii of hCALHM1ΔCt (raspberry), $hCALHM1_{I109W}$ΔCt (light pink) and RuR-$hCALHM1_{I109W}$ΔCt (violet purple) as calculated by the program HOLE[1].

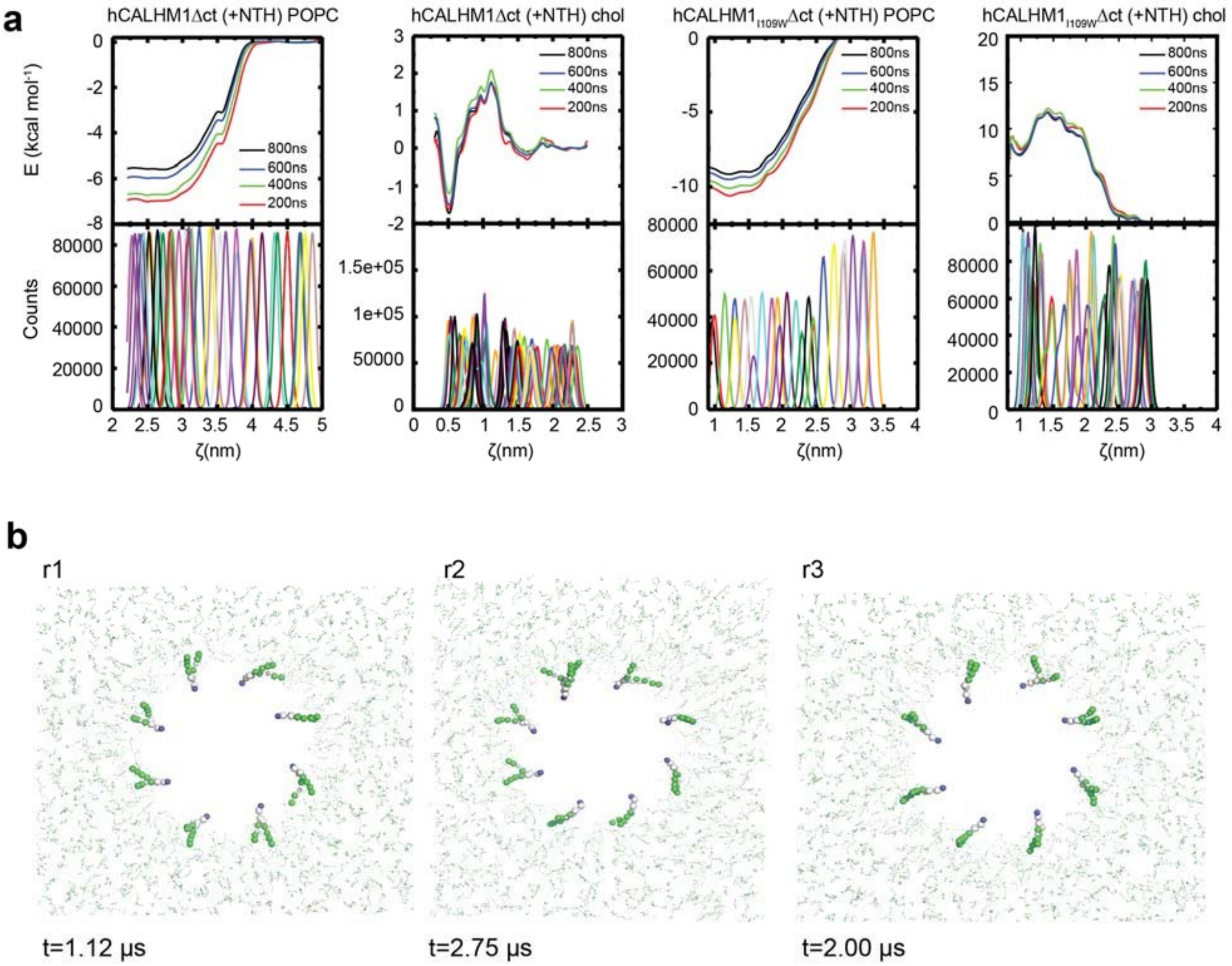

Supplementary Figure 12. Assessment of CG-MD PMF calculation convergence in NTH containing hCALHM1$_{I109W}$Δct and hCALHM1Δct and unbiased CG-MD POPC binding mode. a Block analysis of WT/I109W hCALHM1Δct bound to POPC or cholesterol in increments of 200-400 ns (red), 200-600 ns (green), 200-800 ns (blue), 200-1000 ns (black) demonstrating convergence to within thermal energy for both systems. Associated individual umbrella histograms show good spacing and overlap along all reaction coordinates. **b** Representative snapshots taken from three independent unbiased CG-MD simulations of NTH containing WT hCALHM1Δct where all 8 binding sites have become populated by POPC molecules. In all cases, POPC binds in similar fashion where the choline headgroup is pointed towards the pore and lipidic tails towards the membrane bilayer. The protein, cholesterol and solvent have not been visualized for clarity. Bound POPCs are represented as spheres and unbound POPCs as dots. Spheres and dots are colored according to bead type.

Supplementary Table 1. Cryo-EM data collection, refinement and validation statistics

	hCALHM1Δct (EMD-40231) (PDB-8GMR)	chCALHM1Δct (EMD-40230) (PDB-8GMQ)	hCALHM1 I109WΔct + RR c8 (EMD-40232) (PDB-8S8Z)	hCALHM1 I109WΔct + RR c1 (EMD-40233) (PDB-8S90)	hCALHM1Δct I109W (EMD-40229) (PDB-8GMP)
**Data collection and processing**					
Microscope	Titan Krios	Titan Krios	Titan Krios	Titan Krios	Titan Krios
Camera	K3/counting	K3/counting	K3/counting	K3/counting	K3/counting
Magnification	105,000	105,000	105,000	105,000	105,000
Energy filter	Gatan	Gatan	Gatan	Gatan	Gatan
Energy filter slit width (eV)	20	20	20	20	20
Collection software	EPU	EPU	EPU	EPU	EPU
Voltage (kV)	300	300	300	300	300
Cumulative exposure (e–/Å^2)	60	60	60		60
Exposure rate (e–/Å^2/frame)	2.0	2.0	2.0	2.0	2.0
Defocus range (μm)	0.8 – 2.4	0.8 – 2.4	0.8 – 2.4	0.8 – 2.4	0.8 – 2.4
Pixel size (Å)	0.856	0.856	0.856	0.856	0.856
Symmetry imposed	C8	C8	C8	C1	C8
Number of micrographs	3285	5544	17477	17477	8664
Initial particle images (no.)	660223	1893445	7455143	7455143	2736789
Final particle images (no.)	35412	188439	68351	68351	435473
0.143 FSC half map masked (Å)	3.76	3.36	3.91	4.73	2.80
0.143 FSC half map unmasked(Å)	3.85	3.58	4.4	6.50	3.10
**Refinement**					
Refinement package	Phenix	Phenix	Phenix	Phenix	Phenix
Initial model used (PDB code)	6VAM	6VAM	*hCALHM1Δct*	*hCALHM1Δct*	*hCALHM1Δct*
0.5 FSC model resolution masked (Å)	3.93	3.60	3.9	6.5	2.8
0.5 FSC model resolution unmasked (Å)	4.21	3.80	4.1	6.8	3.0
Model resolution range (Å)	3-6	3-6	3-6	3-7	3-6
Map sharpening *B* factor (Å^2)	-90	-90	-201.5	-216.2	-132.1
Model composition					
Non-hydrogen atoms	13128	15256	16272	15544	14312
Protein residues	1712	1872	2096	2096	1,728
Ligands	POPC	POPC	POPC+RuR	RuR	POPC
CC map vs. model (%)	0.80	0.84	0.84	0.81	0.81
R.m.s. deviations					
Bond lengths (Å)	0.006	0.006	0.011	0.007	0.004
Bond angles (°)	1.222	0.790	1.518	1.217	0.896
Validation					
MolProbity score	2.08	1.96	2.26	2.57	1.91
Clash score	8.53	7.47	13.03	3.64	4.47
Poor rotamers (%)	0	0	0.71	14.30	2.96
Ramachandran plot					
Favored (%)	86.96	90.03	86.15	90.38	95.24
Allowed (%)	12.56	9.97	12.69	9.62	4.76
Outliers (%)	0.48	0	1.15	14.69	0
C-beta deviations	0	0	0	0	0
EMRinger Score	3.92	3.07	2.78	N/A	2.44
CaBLAM outliers (%)	10.40	6.52	8.53	6.98	2.94

Supplementary Table 2. Summary of number of experiments, current density, statistical tests and p values for experiments shown in Figure 3b. *** denotes $p<0.001$ and n.s. denotes “not significant”.

<table>
<tr><td colspan="4">Electrophysiology recordings from hCALHM1Δct wild type and single point mutants</td></tr>
<tr><td>Experiment</td><td colspan="3">Construct Condition</td></tr>
<tr><td rowspan="3">Comparing current density pA/pF</td><td colspan="3">vector (n=9) hCALHM1
(n=10) hCALHM1$_{L67W}$
(n=7) hCALHM1$_{I109W}$
(n=11) hCALHM1$_{V112W}$
(n=5) hCALHM1$_{A116W}$
(n=9) hCALHM1$_{V192W}$
(n=6) hCALHM1$_{T196W}$
(n=10) hCALHM1$_{A199W}$
(n=9)</td></tr>
<tr><td>Statistical test</td><td>Condition</td><td>P value</td></tr>
<tr><td rowspan="9">Unpaired t-test Welch´s correction (two-tailed)</td><td>hCALHM1 (WT vs vector)</td><td>0.0001 (***)</td></tr>
<tr><td rowspan="3"></td><td>hCALHM1 (WT vs Leu67Trp)</td><td>0.9925 (n.s.)</td></tr>
<tr><td>hCALHM1 (WT vs Ile109Trp)</td><td>0.0003 (***)</td></tr>
<tr><td>hCALHM1 (WT vs Val112Trp)</td><td>0.4962 (n.s.)</td></tr>
<tr><td rowspan="3"></td><td>hCALHM1 (WT vs Ala116Trp)</td><td>0.4395 (n.s.)</td></tr>
<tr><td>hCALHM1 (WT vs Val192Trp)</td><td>0.0006 (***)</td></tr>
<tr><td>hCALHM1 (WT vs Thr196Trp)</td><td>0.3810 (n.s)</td></tr>
<tr><td></td><td>hCALHM1 (WT vs Ala199Trp)</td><td>0.3253 (n.s.)</td></tr>
</table>

Supplementary Table 3. Summary of number of experiments, normalized current amplitude, statistical tests and p values for experiments shown in Figure 6b

Whole-cell patch clamp electrophysiology recordings from hCALHM1$_{I109W}$ wild-type with RuR				
Experiment	Statistical test	Condition	Amplitude of control (%)	P value
Effects of RuR	Paired t-test (two-tailed)	hCALHM1$_{I109W}$ 2 µM RuR (n=6)	95.0±1.6	0.0400 (*)
		hCALHM1$_{I109W}$ 10 µM RuR (n=4)	43.1±2.7	0.0004 (***)
		hCALHM1$_{I109W}$ 20 µM RuR (n=7)	7.1±1.1	<0.0001 (****)

Supplementary Table 4. Summary of number of experiments, normalized current amplitude (in %) statistical tests and p values for experiments shown in Figure 6d. ****, ***, **, and * denote 182p<0.0001, p<0.001, p<0.01, and p<0.05, respectively, and n.s. denotes “not significant”.

Whole-cell patch clamp electrophysiology recordings from hCALHM1$_{I109W}$ wild-type and single point mutants				
	Statistical test	Condition	Amplitude (%)	P value
Effects of 20 μM RuR	Paired t-test (two-tailed)	hCALHM1$_{I109W}$ (n=7)	7.1±1.1	<0.0001 (****)
		hCALHM1$_{I109W}$ Gln10Arg (n=4)	79.7±2.3	0.0046 (**)
		hCALHM1$_{I109W}$ Gln13Arg (n=4)	38.9±2.0	0.0001 (***)
		hCALHM1$_{I109W}$ Gln16Arg (n=3)	97.3±1.3	0.2369 (n.s.)
	Statistical test	Condition		P value
	Unpaired t-test Welch´s correction (two-tailed)	hCALHM1$_{I109W}$ (WT vs Gln10Arg)		<0.0001 (****)
		hCALHM1$_{I109W}$ (WT vs Gln13Arg)		0.0001 (***)
		hCALHM1$_{I109W}$ (WT vs Gln16Arg)		<0.0001 (****)

Supplementary References

1. Smart, O. S., Neduvelil, J. G., Wang, X., Wallace, B. A. & Sansom, M. S. P. HOLE: A program for the analysis of the pore dimensions of ion channel structural models. *J. Mol. Graph.* **14**, 354–360 (1996)
