# Supplementary material for: Structure of human CALHM1 reveals key locations for channel regulation and blockade by ruthenium red
Source: Nat Commun. 2023 Jun 28;14:3821. doi: 10.1038/s41467-023-39388-3 (PMC10307800; doi:10.1038/s41467-023-39388-3)
Supplement: Supplementary file 3 — Reporting Summary [file 41467_2023_39388_MOESM3_ESM.pdf]

nature portfolio

Corresponding author(s): Hiro Furukawa

Last updated by author(s): 5/17/2023

Reporting Summary

Nature Portfolio wishes to improve the reproducibility of the work that we publish. This form provides structure for consistency and transparency in reporting. For further information on Nature Portfolio policies, see our Editorial Policies and the Editorial Policy Checklist.

Statistics

For all statistical analyses, confirm that the following items are present in the figure legend, table legend, main text, or Methods section.

n/a	Confirmed	
☐	☒	The exact sample size (n) for each experimental group/condition, given as a discrete number and unit of measurement
☐	☒	A statement on whether measurements were taken from distinct samples or whether the same sample was measured repeatedly
☐	☒	The statistical test(s) used AND whether they are one- or two-sided<br>*Only common tests should be described solely by name; describe more complex techniques in the Methods section.*
☒	☐	A description of all covariates tested
☐	☒	A description of any assumptions or corrections, such as tests of normality and adjustment for multiple comparisons
☐	☒	A full description of the statistical parameters including central tendency (e.g. means) or other basic estimates (e.g. regression coefficient) AND variation (e.g. standard deviation) or associated estimates of uncertainty (e.g. confidence intervals)
☐	☒	For null hypothesis testing, the test statistic (e.g. F, t, r) with confidence intervals, effect sizes, degrees of freedom and P value noted<br>*Give P values as exact values whenever suitable.*
☒	☐	For Bayesian analysis, information on the choice of priors and Markov chain Monte Carlo settings
☒	☐	For hierarchical and complex designs, identification of the appropriate level for tests and full reporting of outcomes
☒	☐	Estimates of effect sizes (e.g. Cohen's d, Pearson's r), indicating how they were calculated

Our web collection on statistics for biologists contains articles on many of the points above.

Software and code

Policy information about availability of computer code

Data collection	EPU 2.10.0.5 was used for Cryo-EM data collection. Clampex 11.2 software was used for electrophysiological data collection.
Data analysis	WARP 1.0.9, cryoSPARC 3.2.0, RELION 3.1, and cisTEM 1.0.2 were used for Cryo-EM single-particle analysis. PHENIX 1.14 and 1.2, Coot 0.893, Chimera 1.14 and Pymol 2.5 were used for model building, refinement and analysis. Clampfit 11.2 and GraphPad Prism 9 were used for electrophysiological data analysis. For MD simulations, MDAnalysis v2.0. , Coot v 0.893, Phenix v1.14, martinize v2.6, Gromacs v. 2021.2 were used.

For manuscripts utilizing custom algorithms or software that are central to the research but not yet described in published literature, software must be made available to editors and reviewers. We strongly encourage code deposition in a community repository (e.g. GitHub). See the Nature Portfolio guidelines for submitting code & software for further information.

Data

Policy information about availability of data

All manuscripts must include a data availability statement. This statement should provide the following information, where applicable:

- Accession codes, unique identifiers, or web links for publicly available datasets
- A description of any restrictions on data availability
- For clinical datasets or third party data, please ensure that the statement adheres to our policy

Previously published cryo-EM models and micrographs used in this study include PBD-6VAM and EMPIAR #10444.

The cryo-EM maps of chCALHM1Δct, hCALHM1Δct, RuR-hCALHM1I109WΔct (c8), RuR-hCALHM1I109WΔct (c1), and hCALHM1I109WΔct generated during this study will be deposited in the Electron Microscopy Data Bank with accession codes: EMD-40230, EMD-40231, EMD-40232, EMD-40233, EMD-40229. Models will be deposited into the Protein Data Bank with accession codes: 8GMQ, 8GMR, 8S8Z, 8S90, 8GMP. The MD simulations data have been deposited to Zenodo. Source data are provided with this paper.

Human research participants

Policy information about studies involving human research participants and Sex and Gender in Research.

Reporting on sex and gender	Not applicable
Population characteristics	Not applicable
Recruitment	Not applicable
Ethics oversight	Not applicable

Note that full information on the approval of the study protocol must also be provided in the manuscript.

Field-specific reporting

Please select the one below that is the best fit for your research. If you are not sure, read the appropriate sections before making your selection.

☒ Life sciences ☐ Behavioural & social sciences ☐ Ecological, evolutionary & environmental sciences

For a reference copy of the document with all sections, see nature.com/documents/nr-reporting-summary-flat.pdf

Life sciences study design

All studies must disclose on these points even when the disclosure is negative.

Sample size	Sample sizes were not predetermined in this study. The sample sizes of Cryo-EM data were governed by the availability of the microscope. Sample sizes were considered sufficient when single particle analysis yielded high resolution features. Electrophysiology experiments were repeated at least three and up to eleven times. Sample size was governed by technical ease of performing patch clamp on specific mutants as well as observation of consistent results from independent experiments.
Data exclusions	In cryo-EM single-particle analysis, particles in 2D and 3D classes which did not possess high-resolution features were removed in the final 3D reconstruction. 3D classification focused around the TMD was done on channel blocker bound samples to exclude the non-ligand bound particles.
Replication	Cryo-EM related experiments including protein expression, purification were reproduced four times independently. Electrophysiology experiments were repeated at least on three and up to eleven different cells. Similar results were observed every time, with the exception of a single outlier for one mutant (shown in Figure 3b). The sample size was increased for this mutant to confirm that it behaved as an outlier. Western blot analyses of surface expression assays were repeated two to three times. Surface expression data showed similar trends in protein expression in every independent experiment. Unbiased CG MD simulations were repeated 3 times with similar results. Atomistic MD simulations were repeated 8-9 times with similar results and PMF calculations were performed once due to computing limitations.
Randomization	In cryo-EM related experiments, all micrograph movies were acquired in random places on the grids and particles were randomly partitioned for resolution and quality assessment in single-particle analysis. Electrophysiology experiments were performed on different batches of cells over different days. Welch's correction was applied to cells analyzed in Figure 3b (effect of point mutations on current density).
Blinding	The investigators were not blinded. Blinding serves no purpose for cryo-EM single-particle analysis. A knowledge of the approximate size and shape of the particles aids in deciding the most optimal data analysis parameters. The cryo-EM data are already randomized and this is considered sufficient. For electrophysiology experiments, surface expression assays, and MD simulations, the experimental outcome for these assays is not affected by whether or not the experimenter is blinded to the result. Furthermore, for MD simulations, a knowledge of the set-up is required to run the experiment.

Reporting for specific materials, systems and methods

We require information from authors about some types of materials, experimental systems and methods used in many studies. Here, indicate whether each material, system or method listed is relevant to your study. If you are not sure if a list item applies to your research, read the appropriate section before selecting a response.

Materials & experimental systems

n/a	Involved in the study
☐	☒ Antibodies
☐	☒ Eukaryotic cell lines
☒	☐ Palaeontology and archaeology
☒	☐ Animals and other organisms
☒	☐ Clinical data
☒	☐ Dual use research of concern

Methods

n/a	Involved in the study
☒	☐ ChIP-seq
☒	☐ Flow cytometry
☒	☐ MRI-based neuroimaging

Antibodies

Antibodies used	Anti-rho-1D4 antibody (University of British Columbia, lot 1015), HRP- conjugated anti-beta-actin antibody (Proteintech, HRP-60008, clone 7D2C10), anti-mouse HRP (Amersham, NA931VS, lot 9653129).
Validation	Sample validation was done by the commercial source. Anti-rho antibody has been validated for bovine and amphibia using western blot, IF and EM (University of British Columbia). HRP-conjugated anti-beta actin antibody has been validated for human, mouse, rat, zebrafish and plant in a variety of cell lines including HEK293 using western blot (Proteintech). Anti-mouse HRP has been validated in HeLa cell lysate for western blotting (Amersham).

Eukaryotic cell lines

Policy information about cell lines and Sex and Gender in Research

Cell line source(s)	HEK293 cells (Thermo), Sf9 cells (Thermo), HEK293 GnTI- cells (ATCC)
Authentication	The cell lines above are not authenicated.
Mycoplasma contamination	All cell lines were confirmed to be negative for mycoplasma.
Commonly misidentified lines (See ICLAC register)	No commonly misidentified cell lines were used in this study.
